# Low-generation dendrimers with a calixarene core and based on a chiral *C*_2_-symmetric pyrrolidine as iminosugar mimics

**DOI:** 10.3762/bjoc.8.107

**Published:** 2012-06-26

**Authors:** Marco Marradi, Stefano Cicchi, Francesco Sansone, Alessandro Casnati, Andrea Goti

**Affiliations:** 1 Dipartimento di Chimica “Ugo Schiff”, Università degli Studi di Firenze, via della Lastruccia 13, I-50019 Sesto Fiorentino (Firenze), Italy; 2 Dipartimento di Chimica Organica e Industriale, Università degli Studi di Parma, Parco Area delle Scienze 17/A, I-43124 Parma, Italy

**Keywords:** calixarenes, cation-responsive system, dendrimers, iminosugars, multivalency

## Abstract

The preparation of low-generation dendrimers based on a simple calix[4]arene scaffold by insertion of the iminosugar-analogue *C*_2_-symmetric 3,4-dihydroxypyrrolidine is described. This methodology allows a rapid incorporation of a considerable number of iminosugar-like moieties in a reduced volume and in a well-defined geometry. The inclusion of alkali-metal ions (sodium and potassium) in the polar cavity defined by the acetamide moieties at the lower rim of the calixarene was demonstrated, which allows also the rigidification of the dendrimer structure and the iminosugar presentation in the clusters. The combination of the supramolecular properties of calixarenes with the advantage of a dendrimeric presentation of repetitive units opens up the possibility of generating well-defined multivalent and multifaceted systems with more complex and/or biologically relevant iminosugars.

## Introduction

Polyhydroxylated pyrrolidines are one of the main classes of naturally occurring sugar mimics [1–2] and belong to the so-called iminosugars [3]. Several iminosugars have shown potential as therapeutics due to the ability to inhibit glycosidases and other enzymes associated with the metabolism of polysaccharides and the processing of glycoproteins [3]. Conjugation of iminosugars onto a polyvalent skeleton has been investigated only occasionally, and their properties as multivalent enzyme inhibitors gave contrasting results. Early findings indicated in fact that poor multivalent phenomena take place [4–6], but more recently moderate [7] to remarkable [8–9] effects have been reported on glycosidases. Unlike lectin-mediated interactions [10], the effect of iminosugar-based multivalent inhibitors on enzyme activity is difficult to rationalise. However, the introduction of several copies of an *N*-alkyl analogue of iminosugar 1-deoxynojirimycin onto a fullerene ball [8] or onto β-cyclodextrin [9] afforded the first pieces of evidence for a significant multivalent effect in glycosidase inhibition. These results highlight the interest in synthesizing multivalent iminosugar-conjugates with well-defined structures. In this context, we aimed at studying the feasibility of combining the supramolecular properties of multivalent scaffolds, such as calixarenes, with the advantages of a dendrimeric presentation of iminosugar analogues.

Calixarenes [11] have been widely employed in host–guest chemistry, first as ligands for small ions and neutral molecules [12–13] and, more recently, for biologically relevant molecules and macromolecules [14]. Multivalent calixarenes functionalised with carbohydrate units (glycocalixarenes) [15] have been extensively reported in the literature and represent examples of sugar clustering on macrocyclic structures [16–17]. Thanks to the “glycoside cluster effect” [18–20], glycocalixarenes can enhance the avidity of interactions between glycans and lectins [15]. Some glycocalixarenes have shown remarkable inhibition properties towards galectins [21–22] or *Pseudomonas Aeruginosa* lectin [23], the inhibition ability being dependent on the macrocyclic conformation and presentation of the glycoside units. With the purpose of expanding the valency and increasing the glycoside density, glycodendrimers have also been synthesised and their properties in protein–carbohydrate interactions have been studied [24–26]. However, the innovative frontier of combining glycodendrimeric arrangements onto a calixarene core has only occasionally been explored. To the best of our knowledge, only one example in which a glycodendrimer was built on a calixarene core has been published [27], while no examples of iminosugar-based calixarene dendrimers have been reported so far.

We report herein the synthesis of low-generation iminosugar-type calixarene-based dendrimers, demonstrating the feasibility of increasing the valency of the cluster by combining a dendrimeric arrangement of iminosugar ligands with a multivalent calixarene core.

## Results and Discussion

**Figure 1:**
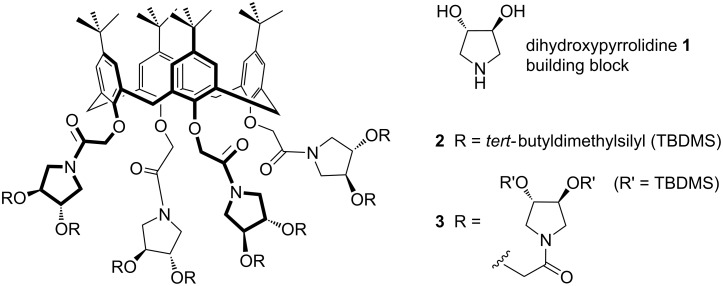
First (**2**) and second (**3**) generation of dendrimers based on chiral *C*_2_-symmetric pyrrolidine **1** and having *p*-*tert-*butyl calix[4]arene as the scaffold.

We chose to address the conjugation of iminosugar-analogue dendrimers, based on the *C*_2_-symmetric (3*S*,4*S*)-3,4-dihydoxypyrrolidine (**1**) unit, to a simple calix[4]arene scaffold (Figure 1). This allows a rapid increase of the valency of the iminosugar dendrimer in a reduced volume. The *C*_2_ symmetry of pyrrolidine **1**, its ready accessibility from the “chiral pool” (L-tartaric acid) [28], and its functional groups (an anchoring amine and two transformable hydroxy groups) make it a convenient chiral AB_2_ building block for the construction of dendrimers [29]. The linkable hydroxy groups of the pyrrolidine rings open up the possibility of constructing calixarene-based dendrimers [30] of higher generation. In addition to its properties as a chiral building block, pyrrolidine **1** may be considered an elemental iminosugar, and it is, by far, easier to handle and to characterise as a consequence of its symmetry. The synthesis of model iminosugar-based calixarene dendrimers may open the way for the construction of more complex systems, decorated with biologically active polyhydroxylated pyrrolidines or piperidines.

**Scheme 1:**
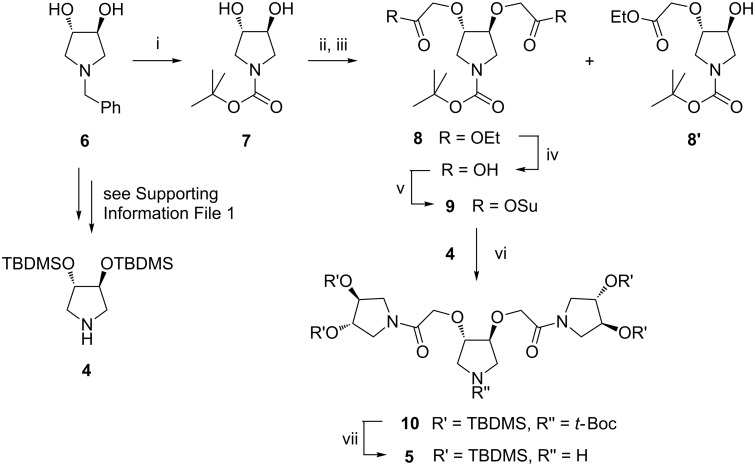
Use of the key intermediate (3*S*,4*S*)-1-benzyl-3,4-dihydroxypyrrolidine (**6**) [31] for the synthesis of pyrrolidine **4** and the pyrrolidine-based dendron **5**. Reagents and conditions: i. Pd(OH)_2_/C, MeOH, HCOO^−^NH_4_^+^, Boc_2_O, reflux, 3 h, 95%; ii. K, THF, 0 °C to rt, 16 h; iii. BrCH_2_CO_2_Et, 4 h, 35% (**8**) and 17% (**8'**); iv. KOH 1 N, EtOH, reflux, 3.5 h, quantitative; v. *N*-hydroxysuccinimide (NHS), AcOEt, dicyclohexylcarbodiimide (DCC), 30 °C, 72 h, 80%, (Su: succinimidyl); vi. *N*,*N*-diisopropylethylamine (DIPEA), CH_2_Cl_2_, 30 °C, 5 d, 77%; vii. trifluoroacetic acid (TFA), CH_2_Cl_2_, rt, 16 h, quantitative.

**Scheme 2:**
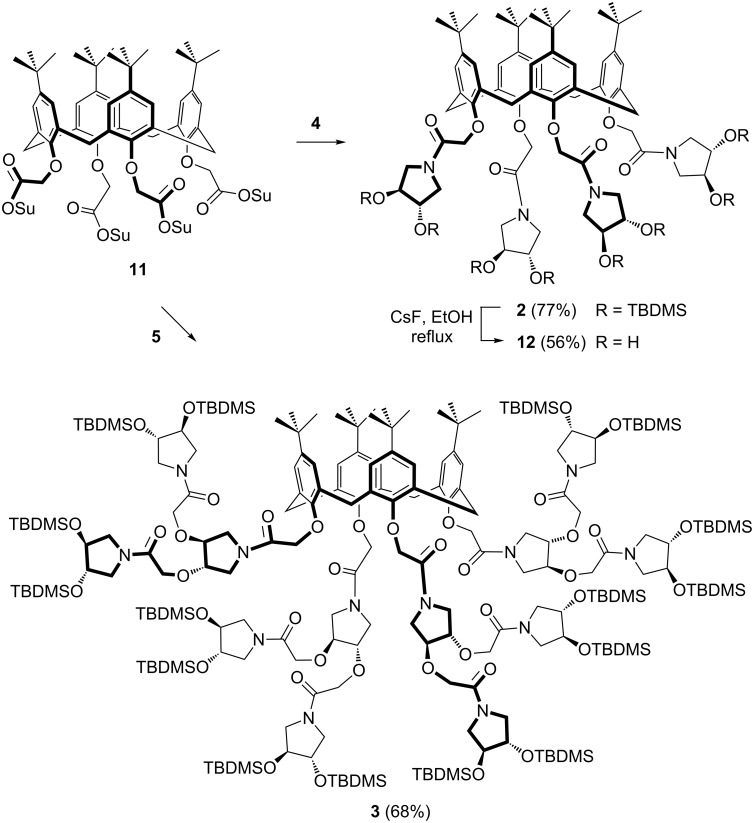
Synthesis of calixarene-based dendrimers **2** and **3**. Reagents and conditions: DIPEA, CH_2_Cl_2_, 30 °C, 5 d, (Su: succinimidyl; TBDMS: *tert-*butyldimethylsilyl).

The synthesis of first- and second-generation iminosugar-based calixarene-dendrimers **2** and **3** (Figure 1) was addressed in order to prove the viability of such a strategy. A convergent synthetic approach was chosen for the conjugation of pyrrolidine **4** and pyrrolidine-based dendron **5** (see Scheme 1) to the calix[4]arene core, via intermediate **11**, in order to obtain iminosugar-based calixarene-dendrimers **2** and **3** (see Scheme 2). The starting calixarene used in this work was the commercially available *p*-*tert-*butyl calix[4]arene, which has the phenolic hydroxy groups at the lower rim and the *tert-*butyl groups at the upper rim. Functionalization of *p*-*tert-*butyl calix[4]arene was performed by standard conversion of the hydroxy groups of the lower rim to activated acetic acid succinimide ester (COOSu) moieties (compound **11**, see Scheme S2 in Supporting Information File 1). These moieties allowed the subsequent conjugation by amide bond formation between **11** and the suitably protected pyrrolidine derivatives **4** and **5** in order to obtain the calixarene-dendrimers **2** and **3**, respectively. The synthesis of (3*S*,4*S*)-3,4-bis(*tert-*butyldimethylsilyloxy)pyrrolidine (**4**) was achieved by following a reported procedure [31], via the key intermediate **6** (Scheme 1 and Supporting Information File 1). Tripyrrolidine **5** required for the second generation calixarene-dendrimer **3** was prepared starting again from the key intermediate **6**, but in this case a modified multistep synthetic procedure was necessary. First, *N*-Boc dihydroxypyrrolidine **7** was obtained from **6** through a “one pot” change of protecting group from benzyl to *tert-*butoxycarbonyl, which occurred in excellent yield by performing the catalytic hydrogenation in methanol under reflux and in the presence of Boc_2_O (Scheme 1). This simple process is unprecedented and may result in a new straightforward method to convert *N*-benzyl amines into *N*-Boc amines once a series of similar compounds are screened. On the other hand, alkylation of the hydroxy groups of **7** with the 2-ethoxycarbonylmethyl linker was problematic. In fact, when sodium hydride was used as a deprotonating agent, no reaction occurred after in situ addition of ethyl bromoacetate. However, a mixture of mono-(**8'**) and di-(**8**) substituted products was obtained by using metallic potassium (Scheme 1). In spite of the poor yield (35%) of the desired product **8**, the starting material **7** and the mono-derivative **8'** could be recovered after column chromatography over silica gel, and used again for the same reaction. The *N*-protected pyrrolidine **8** was then activated for the coupling with pyrrolidine **4** in order to obtain the pyrrolidine-based dendron **5** to be used in assembling the second-generation calixarene-dendrimer **3**. In particular, succinimidyl-activated pyrrolidine **9** was obtained by hydrolysis of ester groups in **8** followed by treatment with DCC/NHS (two steps, 80% overall yield). The amide coupling was then performed by reaction of **9** with pyrrolidine **4** using DIPEA as a base, and this afforded the branched tripyrrolidine **10** in 77% yield after column chromatography over neutral aluminium oxide. The *tert-*butoxycarbonyl group was then removed in the presence of TFA to obtain the pyrrolidine-based dendron **5** (Scheme 1), which was used in the next step without further purification.

Using the SuO-activated calix[4]arene **11** as the scaffold, the planned convergent synthetic approach was completed to afford the first (**2**) and second (**3**) generation iminosugar-based calixarene dendrimers. The coupling between calixarene **11** and pyrrolidine derivatives **4** or **5** was performed as reported before for the synthesis of compound **10** (Scheme 1) and is shown in Scheme 2. The identity of both final compounds **2** and **3**, obtained in good yields (77% and 68%, respectively), was assessed by their spectroscopic data and elemental analysis (see Supporting Information File 1). In particular, integration ratios of the calixarene aromatic (8H, δ ≈ 7 ppm) and *tert*-butyl signals (36H, δ ≈ 1 ppm) versus those of the C*H_3_* of TBDMS at ca. 0 ppm were in good agreement with the number of the expected protons for dendrimers **2** and **3** (48H and 96H, respectively). Thus, an increase of the valency of a model iminosugar dihydroxypyrrolidine **4** in a controlled manner and geometry, by its conjugation to the calixarene scaffold **11** in a dendrimeric fashion, could be demonstrated. Quite interestingly, we could also prove the feasibility of the iminosugar deprotection on the calixarene dendrimer **2**. By treatment with CsF in EtOH, the eight *tert*-butyldimethylsilyl groups could be removed and the deprotected derivative **12** was obtained in 56% yield after trituration with CH_2_Cl_2_ and washing several times with water.

The presence of acetamide moieties at the lower rim of the dendrimers **2** and **3** prompted us to explore the possibility to use alkali metal salts as allosteric effectors in the modulation of the shape and rigidity of the iminosugar presentation by the calixarene scaffold. A common way, in fact, used to rigidify the “mobile cone” structure of tetraalkoxycalix[4]arenes is to introduce strong donating groups, such as amide or ester [32–33], on the phenolic oxygen atoms and an alkali-metal ion. The cation, strongly coordinated by eight oxygen atoms in the polar region created at the lower rim, blocks the calixarene in a “rigid cone” structure [34], also controlling the convergence of the iminosugars.

**Figure 2:**
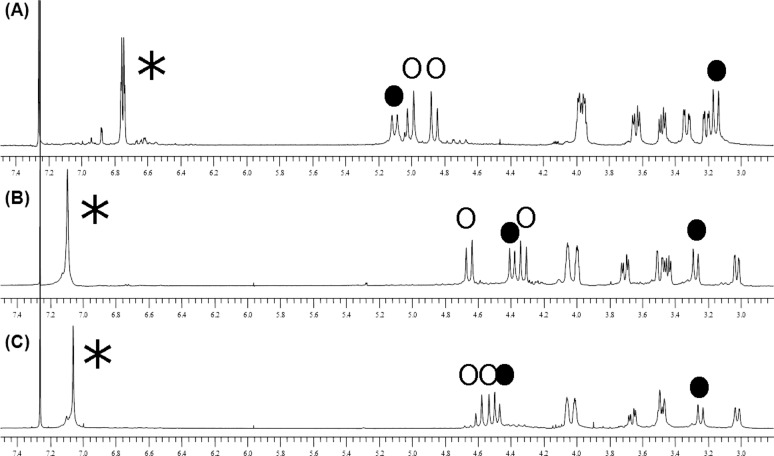
Expansion (about 7 to 3 ppm) of the ^1^H NMR spectra of (A) the free ligand **2**, (B) the sodium picrate complex, and (C) the potassium picrate complex. The full spectra are reported in Supporting Information File 1 (Figure S1). Asterisks: aromatic protons; empty circles: OC*H_2_*CON protons; filled circles: methylene bridge protons.

The ability of first-generation calixarene dendrimer **2** to bind alkali-metal cations was tested by means of NMR, by solid–liquid extraction of solid alkali picrate salts into a CDCl_3_ solution of ligand **2**. A mixture of 0.5 equiv of sodium or potassium picrate and ligand **2** showed the simultaneous presence of the peaks of the complex and of the free ligand indicating a slow exchange regime on the NMR timescale. On the other hand, the NMR analysis of a CDCl_3_ solution of ligand **2** in the presence of an excess of metal picrate (see Figure S1 in Supporting Information File 1) allowed the stoichiometry of the complex to be established. As the picrate salts are scarcely soluble in CDCl_3_, the comparison of the integrals of the picrate signal (a singlet of 2H, around 8.8 ppm) and of the calixarene aromatic protons (a signal of 8H at 7.00–7.10 ppm) indicated that the complexes (both with sodium and potassium) have a 1:1 stoichiometry. As it can be seen in Figure 2, and as reported also for other alkali-metal ion complexes of similar tetramide ligands [35], the cation complexation induces a strong deshielding effect on the aromatic protons of about 0.3 ppm with respect to the free calixarene (as indicated by the asterisks) as a consequence of the electron-withdrawing effect of the metal ion coordinated to the phenolic oxygen.

**Figure 3:**
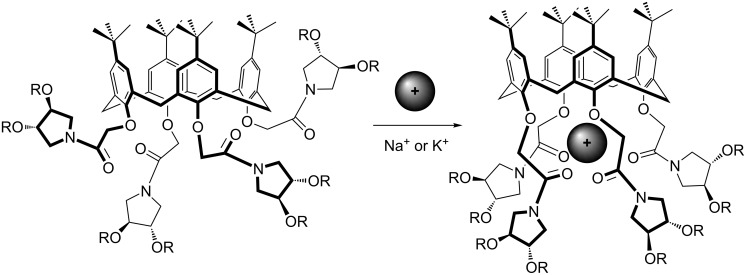
Schematic of the inclusion of alkali-metal ions (sodium and potassium) in the polar cavity defined by the acetamide moieties at the lower rim of the calixarene, and its effect on the rigidification of the calixarene scaffold and organisation of iminosugars.

Quite remarkable is the up-field shift exerted by the OC*H_2_*CON protons (empty circles in Figure 2), which is due to their positioning close to the shielding cone of the aromatic nuclei as a consequence of the C=O coordination to the metal ion (Figure 3). Interesting, and not observed for achiral calixarene tetramide ligands, is the splitting of the OC*H_2_*CON methylene protons into an AB system (empty circles in Figure 2), which, especially in the case of the Na^+^ complex, indicates a quite different chemical environment for the two geminal protons and could be the consequence of a quite twisted square antiprism of coordination around the cation [36]. Consistent shifts are observed for the methylene bridge protons (filled circles in Figure 2), which are in line with previous observations but more difficult to be rationalised also as a consequence of a slight conformational rearrangement of the calixarene scaffold. Less important shifts are obviously observed for the pyrrolidine ring protons, which are quite far from the binding region.

These results show, therefore, that, in spite of the bulky substituents present on the pyrrolidine nuclei, the amide groups can still bind alkali-metal ions quite efficiently. This encourages the use of such compounds not only to study the metal-ion effect on the organisation of iminosugars and on the rigidification of the calixarene scaffold, but also to exploit the ability of these chiral ligands to enantioselectively recognise chiral salts [37] or the ability of their transition-metal complexes to catalyse enantioselective syntheses [38–39], which are objects of current investigations.

## Conclusion

The coupling of low-generation dendrons, based on the chiral *C*_2_-symmetric dihydroxypyrrolidine **1**, with a calix[4]arene scaffold allowed the construction of novel supramolecular architectures, i.e., iminosugar-analogue-based calixarene dendrimers. The convergent synthetic approach used permits the rapid increase of the valency of the iminosugar presentation. Complexation studies with sodium or potassium picrate, carried out on the calixarene-dendrimer **2**, indicated an effective binding of the metal cation at the calixarene lower rim that induces a rigidification of the macrocyclic scaffold and, as a consequence, the convergence of the iminosugars into a restricted region of space. These chiral multivalent dendrimers could thus be interestingly engaged in enantioselective recognition and catalysis. Up to now, pyrrolidine **1** has been used both as a dendrimeric building block and iminosugar model; its substitution at the hydroxy groups with more complex and biologically active iminosugars, such as polyhydroxylated pyrrolidines, would also allow the investigation of the corresponding calixarene dendrimers in interactions with enzymatic receptors.

## Supporting Information

File 1 Experimental procedures; spectroscopic and analytical data.

## References

[1] Asano N, Nash R J, Molyneux R J, Fleet G W J (2000). Tetrahedron: Asymmetry.

[2] Elbein A D (1987). Annu Rev Biochem.

[3] Compain P, Martin O R (2007). Iminosugars: From Synthesis to Therapeutic Applications.

[4] Johns B A, Johnson C R (1998). Tetrahedron Lett.

[5] Lohse A, Jensen K B, Lundgren K, Bols M (1999). Bioorg Med Chem.

[6] Wennekes T, van den Berg R J B H N, Bonger K M, Donker-Koopman W E, Ghisaidoobe A, van der Marel G A, Strijland A, Aerts J M F G, Overkleeft H S (2009). Tetrahedron: Asymmetry.

[7] Diot J, García-Moreno M I, Gouin S G, Ortiz Mellet C, Haupt K, Kovensky J (2009). Org Biomol Chem.

[8] Compain P, Decroocq C, Iehl J, Holler M, Hazelard D, Mena Barragán T, Ortiz Mellet C, Nierengarten J-F (2010). Angew Chem, Int Ed.

[9] Decroocq C, Rodríguez-Lucena D, Russo V, Mena Barragán T, Ortiz Mellet C, Compain P (2011). Chem–Eur J.

[10] Kiessling L L, Gestwicki J E, Strong L E (2006). Angew Chem, Int Ed.

[11] Gutsche C D (2008). Calixarenes: An Introduction.

[12] Ungaro R, Arduini A, Casnati A, Pochini A, Ugozzoli F (1996). Pure Appl Chem.

[13] Casnati A, Sansone F, Ungaro R, Gokel G W (2003). Calixarene Receptors in Ion Recognition and Sensing. Advances in Supramolecular Chemistry.

[14] Sansone F, Baldini L, Casnati A, Ungaro R (2010). New J Chem.

[15] Marra A, Scherrmann M-C, Dondoni A, Ungaro R, Casnati A, Minari P (1995). Angew Chem, Int Ed Engl.

[16] Dondoni A, Marra A (2010). Chem Rev.

[17] Sansone F, Rispoli G, Casnati A, Ungaro R, Renaudet O, Spinelli N (2011). Multivalent Glycocalixarenes. Synthesis and Biological Applications of Glycoconjugates.

[18] Lee Y C, Lee R T (1995). Acc Chem Res.

[19] Kiessling L L, Pohl N L (1996). Chem Biol.

[20] Lundquist J J, Toone E J (2002). Chem Rev.

[21] André S, Sansone F, Kaltner H, Casnati A, Kopitz J, Gabius H-J, Ungaro R (2008). ChemBioChem.

[22] André S, Grandjean C, Gautier F-M, Bernardi S, Sansone F, Gabius H-J, Ungaro R (2011). Chem Commun.

[23] Cecioni S, Lalor R, Blanchard B, Praly J-P, Imberty A, Matthews S E, Vidal S (2009). Chem–Eur J.

[24] Chabre Y M, Roy R (2010). Adv Carbohydr Chem Biochem.

[25] Turnbull W B, Stoddart J F (2002). Rev Mol Biotechnol.

[26] Röckendorf N, Lindhorst T K, Vögtle F, Schalley C A (2001). Glycodendrimers. Dendrimers IV. Metal Coordination, Self Assembly, Catalysis.

[27] Roy R, Kim J M (1999). Angew Chem, Int Ed.

[28] Nagel U, Kinzel E, Andrade J, Prescher G (1986). Chem Ber.

[29] Cicchi S, Goti A, Rosini C, Brandi A (1998). Eur J Org Chem.

[30] Baklouti L, Cheriaa N, Mahouachi M, Abidi R, Kom J S, Kim Y, Vicens J (2006). J Inclusion Phenom Macrocyclic Chem.

[31] Arakawa Y, Yoshifuji S (1991). Chem Pharm Bull.

[32] Arduini A, Ghidini E, Pochini A, Ungaro R, Andreetti G D, Calestani G, Ugozzoli F (1988). J Inclusion Phenom Mol Recognit Chem.

[33] Arnaud-Neu F, Barboso S, Berny F, Casnati A, Muzet N, Pinalli A, Ungaro R, Schwing-Weill M-J, Wipff G (1999). J Chem Soc, Perkin Trans 2.

[34] Arduini A, Fabbi M, Mantovani M, Mirone L, Pochini A, Secchi A, Ungaro R (1995). J Org Chem.

[35] Arduini A, Pochini A, Reverberi S, Ungaro R (1986). Tetrahedron.

[36] Casnati A, Cavallo G, Metrangolo P, Resnati G, Ugozzoli F, Ungaro R (2009). Chem–Eur J.

[37] Yakovenko A V, Boyko V I, Kalchenko V I, Baldini L, Casnati A, Sansone F, Ungaro R (2007). J Org Chem.

[38] Casolari S, Cozzi P G, Oriolo P, Tagliavini E, Umani-Ronchi A (1997). J Chem Soc, Chem Commun.

[39] Pinkhassik E, Stibor I, Casnati A, Ungaro R (1997). J Org Chem.

