# Supplementary material for: Low-generation dendrimers with a calixarene core and based on a chiral C2-symmetric pyrrolidine as iminosugar mimics
Source: Beilstein J Org Chem. 2012 Jun 26;8:951–7. doi: 10.3762/bjoc.8.107 (PMC3388885; doi:10.3762/bjoc.8.107)
Supplement: File 1 — Experimental procedures; spectroscopic and analytical data. [file Beilstein_J_Org_Chem-08-951-s001.pdf]

Supporting Information

for

Low-generation dendrimers with a calixarene core and based on a chiral C_2-symmetric pyrrolidine as iminosugar mimics

Marco Marradi*[1,§], Stefano Cicchi[1], Francesco Sansone[2], Alessandro Casnati[2] and Andrea Goti[1]

Address: [1]Dipartimento di Chimica "Ugo Schiff", Università degli Studi di Firenze, via della Lastruccia 13, I-50019 Sesto Fiorentino (Firenze), Italy and [2]Dipartimento di Chimica Organica e Industriale, Università degli Studi di Parma, Parco Area delle Scienze 17/A, I-43124 Parma, Italy

Email: Marco Marradi* - mmarradi.ciber-bbn@cicbiomagune.es; Stefano Cicchi - stefano.cicchi@unifi.it; Francesco Sansone - francesco.sansone@unipr.it; Alessandro Casnati - casnati@unipr.it; Andrea Goti - andrea.goti@unifi.it

* Corresponding author

[§]present address: Laboratory of GlycoNanotechnology, Biofunctional Nanomaterials Unit, CIC biomaGUNE / CIBER-BBN, Pº Miramón 182, San Sebastián E-20009, Spain

Experimental procedures; spectroscopic and analytical data

1. General remarks

All chemicals were purchased as reagent grade from Sigma-Aldrich and used without further purification. All reactions requiring anhydrous conditions were carried out under nitrogen and solvents were appropriately dried before use. R_f values refer to TLC on 0.25 mm silica gel plates (Merck F_{254}). Melting points (mp) were determined on an RCH Kofler apparatus. Optical rotation measurements were performed (at the equilibrium for tautomeric compounds) with a Jasco DIP-370 polarimeter. ^{1}H and ^{13}C NMR spectra were recorded at 200 MHz and 50.3 MHz, respectively, with a Varian Gemini spectrometer, or at 400 MHz and 100 MHz, respectively, with a Varian MERCURY plus spectrometer (where specified in brackets) in $CDCl_3$ solution, unless otherwise stated. Chemical shifts for ^{1}H and ^{13}C NMR spectra are given in ppm and the coupling constants values (*J*) are given in Hertz. IR spectra were recorded with a BX FT-IR Perkin-Elmer System spectrophotometer (cm^{-1}). Mass spectra were recorded with a QMD 1000 Carlo Erba instrument (EI, 70 eV) after direct inlet (relative percentages are given in brackets). Elemental analyses were performed with a Perkin-Elmer 240 C instrument.

The synthesis of the key intermediate **6** and pyrrolidine **4** used to construct the first-generation calixarene dendrimer **2** was performed by following a reported procedure [1S], as depicted in Scheme S1, with minor modifications.

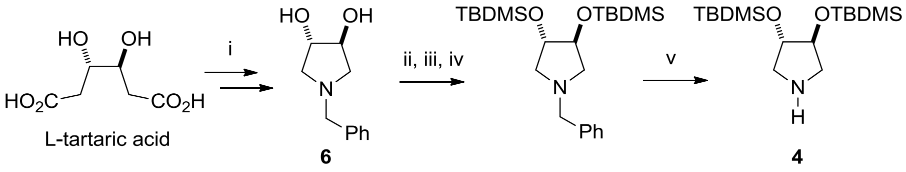

Scheme S1: Synthesis of *O*-protected 3,4-dihydroxypyrrolidine starting from L-tartaric acid. Reagents and conditions: i. see [1S]; ii. Imidazole, DMF, r.t., 5 min; iii. TBDMSCl, 0 °C, 1h; iv. 60 °C, 12 h, 99%; v. $H_2/Pd(OH)_2/C$, $MeOH/CH_2Cl_2$, r. t., 12 h, 99%.

The preparation of SuO-activated calixarene **11** was carried out as depicted in Scheme S2. Alkylation of commercially available *p*-*tert*-butyl calix[4]arene with ethyl bromoacetate was carried out by following a reported protocol [2S]. The ethyl ester of *p*-*tert*-butyl calix[4]arene was then hydrolyzed to the corresponding acid [3S], which was finally activated with NHS [4S] to obtain **11**.

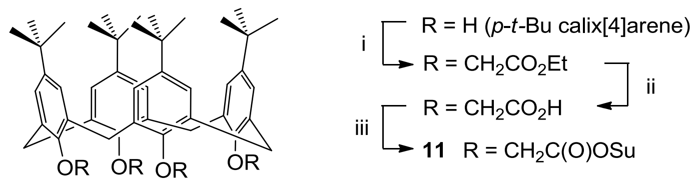

Scheme S2: Functionalization of *p*-*tert*-butyl calix[4]arene. Reagents and conditions: i. $BrCH_2CO_2Et$, acetone, K_2CO_3, 56 °C, 22 h, 98%; ii. KOH 1N, EtOH, reflux, 3.5 h, quantitative; iii. NHS, AcOEt, DCC, 30 °C, 72 h, 80%.

2. Synthesis of pyrrolidine derivatives

(3*S*,4*S*)-1-Benzyl-3,4-bis-(*tert*-butyldimethylsilanyloxy)pyrrolidine [1S]

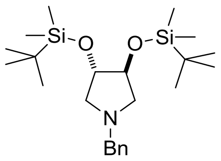

Imidazole (3.5 g, 51.4 mmol) was added under a nitrogen atmosphere to a stirred solution of (3*S*,4*S*)-1-benzyl-3,4-dihydroxypyrrolidine (**6**) [1S] (2.5 g, 12.9 mmol) in dry DMF (30 mL) and then *tert*-butyldimethylsilyl chloride (4.3 g, 28.5 mmol) was added in portions at 0 °C. The solution was stirred for 1 h at this temperature and then left at 60 °C overnight (massive salts precipitation). The mixture was diluted with water (30 mL) and washed with petroleum ether (3 × 30 mL). The organic phase was washed again with water (2 × 30 mL), filtered, washed with petroleum ether, and dried over anhydrous Na_2SO_4. After

concentration under reduced pressure, the desired product (5.4 g, 12.8 mmol) was obtained.

Colorless oil, 99% yield; ^{1}H NMR δ 7.33–7.29 (m, 5H), 4.11 (t, 2H, *J* = 4.4 Hz), 3.62 (m, 2H, AB system), 2.85 (dd, 2H, *J* = 9.5, 4.4 Hz), 2.45 (dd, 2H, *J* = 9.5, 4.4 Hz), 0.87 (s, 18H), 0.05 (s, 6H), 0.05 (s, 6H).

(3*S*,4*S*)-3,4-Bis(*tert*-butyldimethylsilyloxy)pyrrolidine (4) [1S]

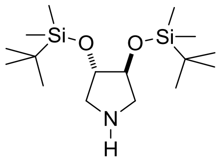

Palladium hydroxide 40% on activated carbon (750 mg) was added to a stirred solution of (3*S*,4*S*)-1-benzyl-3,4-bis-(*tert*-butyldimethylsilanyloxy)pyrrolidine (1.5 g, 3.56 mmol) in MeOH (18 mL) and CH_2Cl_2 (1 mL) under a nitrogen atmosphere. The mixture was stirred overnight at rt under a hydrogen atmosphere. The catalyst was filtered over Celite, washing with CH_2Cl_2, and the dark solution concentrated under reduced pressure to afford the desired product **4** (1.18 g, 3.56 mmol).

Foaming solid, quantitative; ^{1}H NMR δ 6.71 (bs, 1H, exchanging proton), 4.03 (m, 2H), 3.33 (dd, 2H, *J* = 11.7, 3.7 Hz), 2.98 (d, 2H, *J* = 11.7 Hz), 0.85 (s, 18H), 0.05 (s, 12H).

(3*S*,4*S*)-1-*tert*-Butoxycarbonyl-3,4-bis(dihydroxy)pyrrolidine (7) [5S]

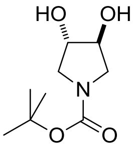

(3*S*,4*S*)-1-benzyl-3,4-dihydroxypyrrolidine [1S] (2.5 g, 193.24 g·mol^{-1}, 12.9 mmol), di-*tert*-butyldicarbonate (4.2 g, 218.25 g·mol^{-1}, 19.3 mmol), ammonium formate (3.7 g, 63.06 g·mol^{-1}, 58 mmol) and palladium hydroxide 40% on activated carbon were combined in methanol (45 mL). The mixture was heated under reflux for 3 h, filtered over Celite and concentrated under reduced pressure to give a yellow solid, which was purified by flash column chromatography (FCC) (gradient AcOEt/MeOH, starting with AcOEt, gradient with 2% methanol) to obtain **7** (2.5 g, 12.3 mmol).

White solid, 95% yield (AcOEt, R_f = 0.21); ^{1}H NMR δ 4.18 (bs, 2H), 3.66 (dd, 2H, *J* = 11.7, 3.7 Hz), 3.44–3.30 (bs, 2H), 1.46 (s, 9H).

(3*S*,4*S*)-1-*tert*-Butoxycarbonyl-3,4-bis(ethoxycarbonylmethoxy)pyrrolidine (8)

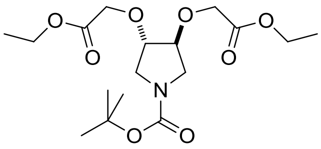

Diol **7** (203 mg, 1.0 mmol) was dissolved in dry THF (5 mL) and cooled in an ice bath. Potassium was then carefully added under vigorous stirring, and a precipitate formed. The mixture was left under stirring and allowed to reach rt, and then kept under a nitrogen atmosphere overnight. The next day, in the presence of residual potassium, ethyl bromoacetate (400 µL, 3.6 mmol) was added until the metal disappeared and a yellowish slurry was formed. After 4 h

the reaction mixture was diluted with AcOEt (10 mL) and carefully filtered, and the resulting mixture was washed with water (2 × 10 mL). The organic phase was concentrated under reduced pressure to give a yellow oil, which was purified by FCC over silica gel using as eluent AcOEt/petroleum ether = 1:3. After the elution of the ethyl bromoacetate in excess, diprotected derivative **8** (130 mg, 0.35 mmol) was obtained with a small amount of monoether **8'** (50 mg, 289.32 g·mol^{-1}, 0.17 mmol).

Major product 8 (di-substituted): Oil, 35% yield (AcOEt/petroleum ether = 1:3, R_f = 0.61); ^{1}H NMR (400 MHz) δ 4.22 (q, 4H, *J* = 7.3 Hz), 4.17–4.07 (m, 6H), 3.60 (dd, 2H, *J* = 12.1, 4.4 Hz), 3.50 (bd, 1H, *J* = 12.1 Hz), 3.43 (bd, 1H, *J* = 12.1 Hz), 1.46 (s, 9H), 1.29 (t, 6H, *J* = 7.3 Hz); ^{13}C NMR δ 169.9 (s, 1C), 154.3 (s, 2C), 82.2 and 81.0 (d, 2C, two rotamers), 79.5 (s, 1C), 66.9 and 66.7 (t, 2C, two rotamers), 61.0 (t, 2C), 49.5 and 48.5 (t, 2C, two rotamers), 28.4 (q, 3C), 14.1 (q, 2C); IR (CH_2Cl_2): 2981 (m), 2876 (w), 1752 (s), 1690 (s), 1410 (s), 1213 (m), 1132 (s) cm^{-1}; MS *m*/*z* (% relative intensity) 375 (2), 363 (18), 178 (14), 143 (21), 135 (86), 91 (100), 77 (44); Anal. calcd. for $C_{17}H_{29}NO_8$: C, 54.39; H, 7.79; N, 3.73; found: C, 54.32; H, 7.87; N, 3.40; $[\alpha]_D^{22}$ = +17 (*c* = 0.35, CH_2Cl_2).

Minor product 8' (mono-substituted): Oil, 17% yield (AcOEt/petroleum ether = 1:3, R_f = 0.31); ^{1}H NMR δ 4.28 (bs, 1H), 4.19 (q, 2H, *J* = 7.3 Hz), 4.16–4.07 (m, 2H), 3.93–3.82 (m, 1H), 3.74–3.67 (m, 1H), 3.60 (dd, 2H, *J* = 11.7, 5.0 Hz), 3.40–3.23 (m, 2H), 1.41 (s, 9H), 1.25 (t, 3H, *J* = 7.3 Hz); ^{13}C NMR δ 166.6 (s, 1C), 154.5 (s, 1C), 84.2 and 83.6 (d, 1C, two rotamers), 79.6 (s, 1C), 73.6 and 72.6 (d, 1C, two rotamers), 66.9 (t, 1C), 61.1 (t, 1C), 51.4 and 51.3 (t, 1C, two rotamers), 49.3 and 48.3 (t, 1C, two rotamers), 28.5 (q, 3C), 14.1 (q,

1C); Anal. calcd. for $C_{13}H_{23}NO_6$: C, 53.97; H, 8.01; N, 4.84; found: C, 53.08; H, 7.85; N, 4.73.

(3*S*,4*S*)-1-*tert*-Butoxycarbonyl-3,4-bis(carboxymethoxy)pyrrolidine

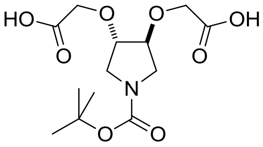

KOH 1 M (41 mg, 0.72 mmol) was added to a stirred suspension of **8** (51 mg, 0.14 mmol) in EtOH (3 mL). The mixture was heated for 3.5 h under reflux and then cooled to 0 °C. HCl 1 M was dropwise added until the pH was less than 4, and the product was extracted with AcOEt (10 mL) and CH_2Cl_2 (2 × 10 mL). The combined organics were dried over anhydrous Na_2SO_4. After concentration under reduced pressure, the desired product was obtained (45 mg, 0.14 mmol) and used in the next step without further purification.

Colorless oil, quantitative; ^{1}H NMR δ 8.53 (bs, 2H), 4.30–4.10 (m, 6H), 3.73–3.31 (m, 4H), 1.44 (s, 9H); ^{13}C NMR δ 173.9 (s, 2C), 155.1 (s, 1C), 82.1 and 81.0 (d, 2C, two rotamers), 80.6 (s, 1C), 66.5 (bt, 2C), 49.5 and 48.6 (t, 2C, two rotamers), 28.4 (s, 3C).

(3*S*,4*S*)-1-*tert*-Butoxycarbonyl-3,4-bis(succinimidoxycarbonylmethoxy)pyrrolidine (9)

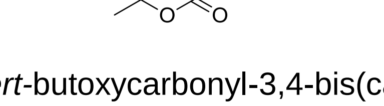

A solution of (3*S*,4*S*)-1-*tert*-butoxycarbonyl-3,4-bis(carboxymethoxy)pyrrolidine (45 mg, 0.14 mmol) in AcOEt (2 mL, freshly distilled from P_2O_5) was added to a

stirred suspension of NHS (34 mg, 0.30 mmol) in dry AcOEt (3 mL). A solution of DCC (62 mg, 0.30 mmol) in dry AcOEt (3 mL) was then added. The mixture was stirred at 30 °C over the weekend. The formed slurry was filtered over Celite, washing several times with AcOEt. The clear solution was concentrated under reduced pressure to compound **9** (71 mg, 0.14 mmol) and used in the next step without further purification.

Solid, quantitative; ^{1}H NMR δ 4.61–4.39 (m, 4H), 4.24–4.15 (m, 2H), 3.60 (dd, 2H, J = 12.5, 4.4 Hz), 3.44 (t, 2H, J = 12.5 Hz), 2.83 (s, 8H), 1.43 (s, 9H).

Branched tripyrrolidine 10

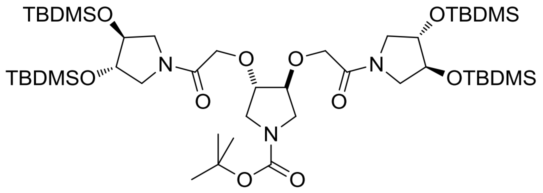

A solution of pyrrolidine **4** (102 mg, 0.31 mmol) and DIPEA (106 µL, 0.62 mmol) in dry CH_2Cl_2 (4 mL) was added to a solution of crude **9** (100 mg, 0.14 mmol) in CH_2Cl_2 (1.5 mL). After 5 d at 30 °C under a nitrogen atmosphere CH_2Cl_2 (20 mL) was added and the organic phase was washed with water (3 × 20 mL). The clear solution was concentrated under reduced pressure and the crude was purified by FCC (gradient CH_2Cl_2/MeOH, starting with pure CH_2Cl_2) over neutral aluminium oxide (activated with 2% of water) to obtain **10** (275 mg, 0.29 mmol).

Oil, 95% yield (CH_2Cl_2/MeOH = 100:5, R_f = 0.33); ^{1}H NMR δ 4.28–3.97 (m, 10H), 3.65–3.38 (m, 10H), 3.24–3.18 (m, 2H), 1.43 (s, 9H), 0.85 (s, 36H), 0.06 (s, 24H); ^{13}C NMR δ 167.8 (s, 2C), 154.5 (s, 1C), 81.9 and 80.6 (d, 4C, two rotamers), 79.4 (s, 1C), 76.7 and 74.6 (d, 2C, two rotamers), 68.6 and 68.2 (t, 2C, two rotamers), 52.1 and 51.9 (t, 4C, two rotamers), 49.8 and 48.7 (t, 2C,

two rotamers), 28.5 (q, 3C), 25.6 (q, 12C), 17.9 (s, 4C), −4.9 (q, 8C); IR (CH_2Cl_2): 2957 (m), 2929 (m), 2856 (w), 1685 (s), 1657 (s), 1472 (m), 1450 (m), 1415 (s), 1252 (m), 1112 (s) cm^{-1}; MS *m*/*z* (% relative intensity) 900 (6), 706 (8), 671 (5), 615 (5), 581 (8), 580 (12), 269 (5), 268 (8), 237 (6), 236 (11), 178 (19), 177 (30), 174 (6), 173 (9), 149 (64), 129 (89), 56 (100); Anal. calcd. for $C_{45}H_{91}N_3O_{10}Si_{14}$: C, 57.10; H, 9.69; N, 4.44; found: C, 57.09; H, 9.55; N, 4.39; $[\alpha]_D^{22}$ = −2.5 (*c* = 0.585, CH_2Cl_2).

Trifluoracetic acid salt of pyrrolidine-based dendron 5

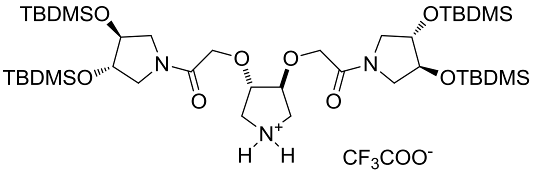

TFA (100 μL, 1.3 mmol) was carefully added under a nitrogen atmosphere to a cooled (0 °C) and stirred suspension of compound **10** (75 mg, 0.08 mmol) in dry CH_2Cl_2 (1 mL). The resulting solution was stirred for one night and then evaporated to afford the desired salt **5**, which was used in the next step without further purification.

Oil, quantitative (CH_2Cl_2/MeOH = 100:1, R_f = 0.19); ^1H NMR δ ≈10.70–9.80 (bs, 1H, exchanging proton), 9.42–9.11 (bs, 1H, exchanging proton), 4.47–3.99 (m, 10H), 3.76–3.34 (m, 10H), 3.21–3.08 (m, 2H), 0.85 (s, 36H), 0.06 (s, 24H).

3. Synthesis of calix[4]arene derivatives

25,26,27,28-Tetrakis(ethoxycarbonylmethoxy)-*p*-*tert*-butylcalix[4]arene [2S]

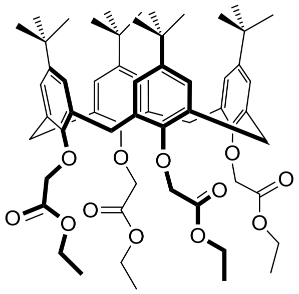

Ethyl bromoacetate (17.7 mL, 160 mmol) was added to a stirred suspension of *p*-*tert*-butylcalix[4]arene (5.2 g, 8.1 mmol) in acetone (240 mL) in the presence of anhydrous potassium carbonate (22 g, 160 mmol). The mixture was heated gently for 22 h under reflux. Acetone was removed under reduced pressure and the resulting slurry was partitioned between water (150 mL) and AcOEt, which is used for the extraction (3 × 100 mL). The organic layer was washed with water (3 × 100 mL) and dried over anhydrous Na_2SO_4. After concentration a yellow oil was obtained. The excess of ethyl bromoacetate was distilled off under high vacuum. The resulting syrup was allowed to rest overnight at rt to form a solid mass. The solid was triturated in hot EtOH, and once the solid was deposited the yellow solution was taken off with a Pasteur pipette (this procedure was repeated three times). Finally the solid was filtered with a Hirsch funnel, washing with cold EtOH, and kept under vacuum in a desiccator using $CaCl_2$ as the drying agent. The crude product (7.8 g, 7.9 mmol) was used in the next step without further purification.

White solid, 98% yield, (AcOEt/petroleum ether = 1:3, R_f = 0.21); 0.35 (AcOEt); ^{1}H NMR δ 6.77 (s, 8H), 4.85 (d, 4H, *J* = 13.1 Hz), 4.81 (s, 8H), 4.20 (q, 8H, *J* = 7.3 Hz), 3.20 (d, 4H, *J* = 13.1 Hz), 1.29 (t, 12H, *J* = 7.3 Hz), 1.07 (s, 36H).

25,26,27,28-Tetrakis(carboxymethoxy)-*p*-*tert*-butylcalix[4]arene [2S,3S]

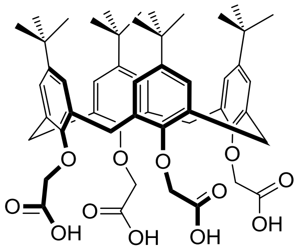

KOH 1 M (1.74 g, 31 mmol) was added to a stirred suspension of 25,26,27,28-tetrakis(ethoxycarbonylmethoxy)-*p*-*tert*-butylcalix[4]arene (3 g, 3 mmol) in EtOH (60 mL). The mixture was heated for 3.5 h under reflux (at about 100 °C the solution became completely clear) and then cooled to 0 °C. As HCl 2 M (90 mL) was added a massive precipitation occured. The solid was filtered with a Hirsch funnel, washing with cold water, and kept under vacuum in a desiccator using $CaCl_2$ as the drying agent. The crude (2.64 g, 3 mmol) was used in the next step without further purification.

White solid, quantitative; ^{1}H NMR δ ≈9.50–7.30 (bs, 4H), 6.90 (s, 8H), 4.63 (s, 8H), 4.63 (m, 4H), 3.25 (d, 4H, *J* = 12.5 Hz), 1.10 (s, 36H).

25,26,27,28-Tetrakis(succinimidoxycarbonylmethoxy)-*p*-*tert*-butylcalix[4]arene (11) [4S]

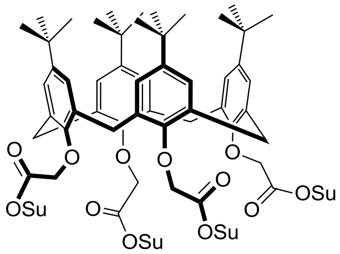

25,26,27,28-Tetrakis(carboxymethoxy)-*p*-*tert*-butylcalix[4]arene (881 mg, 1.0 mmol) was added to a stirred suspension of NHS (480 mg, 4.2 mmol) in AcOEt (3 mL, freshly distilled from P_2O_5). A solution of DCC (870 mg, 4.2 mmol)

in dry AcOEt (3 mL) was then added. The colour changed to yellow. The mixture was stirred at 30 °C for 60 h. The formed slurry was filtered over Celite, washing with AcOEt. The clear solution was concentrated under reduced pressure to compound **11** (1.03 g, 0.80 mmol), which was used in the next step without further purification.

White solid, 80% yield; ^{1}H NMR δ 6.79 (s, 8H), 5.16 (s, 8H), 4.80 (d, 4H, *J* = 13.2 Hz), 3.25 (d, 4H, *J* = 13.2 Hz), 2.84 (s, 16H), 1.08 (s, 36H).

First generation calixarene dendrimer

25,26,27,28-Tetrakis((3'*S*,4'*S*)-bis-(*tert*-butyl-dimethyl-silyloxy)-pyrrolidinylcarbonylmethoxy)-*p*-*tert*-butylcalix[4]arene (2)

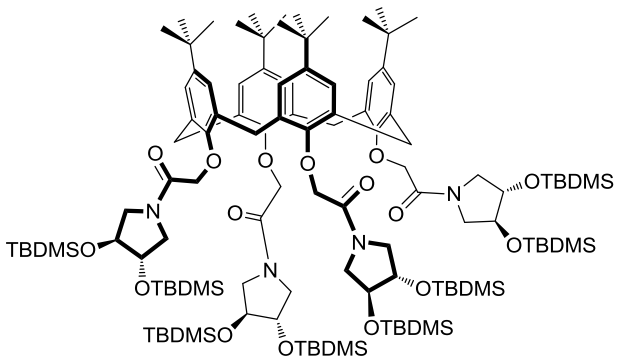

A solution of pyrrolidine **4** (146 mg, 0.44 mmol) and DIPEA (150 µL, 0.88 mmol) in dry CH_2Cl_2 (2 mL) was added to a solution of **11** (127 mg, 0.1 mmol) in CH_2Cl_2 (1 mL). After 5 d at 30 °C under a nitrogen atmosphere the solution became dark. CH_2Cl_2 (20 mL) was then added and the organic phase was extensively washed with milli-Q water (6 × 20 mL). The clear solution was concentrated under reduced pressure to a dark oil, which was purified by FCC (gradient CH_2Cl_2/MeOH, starting with CH_2Cl_2/MeOH = 99:1) over neutral aluminium oxide (activated with 2% of water) to obtain the first generation silylated calixarene dendrimer **2** (165 mg, 0.077 mmol).

White solid, 77% yield (CH_2Cl_2/MeOH = 95:5, R_f = 0.29); mp 105 °C; ^{1}H NMR (400 MHz) δ 6.76–6.74 (m, 8H), 5.10 (d, 4H, J = 12.6 Hz), 4.94 (AB system, 8H), 3.99–3.94 (m, 8H), 3.64 (dd, 4H, J = 12.2, 4.9 Hz), 3.48 (dd, 4H, J = 10.7, 4.9 Hz), 3.33 (dd, 4H, J = 12.2, 2.9 Hz), 3.21 (dd, 4H, J = 10.7, 2.9 Hz), 3.15 (d, 4H, J = 12.6 Hz), 1.05 (s, 36H), 0.87 (s, 72H), 0.07 (s, 12H), 0.06 (s, 24H), 0.05 (s, 12H); ^{13}C NMR δ 169.3 (s, 4C), 154.0 (s, 4C), 144.3 (s, 4C), 134.0 (s, 4C), 133.6 (s, 4C), 125.4 (d, 4C), 124.9 (d, 4C), 76.9 (d, 4C), 75.1 (d, 4C), 72.2 (t, 4C), 51.3 (t, 8C), 33.7 (s, 4C), 32.5 (t, 4C), 31.4 (s, 12C), 25.8 (q, 24C), 18.0 (s,

4C), 17.8 (s, 4C), −4.6 (q, 8C), −4.8 (q, 8C); IR (KBr): 3449 (s), 3002 (w), 2956 (m), 2931 (m), 2859 (w), 1664 (s), 1474 (m), 1258 (m) 1114 (m), 837 (s) cm^{-1}; Anal. calcd. for $C_{116}H_{204}N_4O_{16}Si_8$: C, 65.24; H, 9.63; N, 2.62; O, 11.99; Si, 10.52; found: C, 64.99; H, 9.59; N, 2.66; $[\alpha]_D^{24}$ = +16 (c = 1.05, CH_2Cl_2).

25,26,27,28-Tetrakis((3'*S*,4'*S*)-bis(dihydroxy)-pyrrolidinylcarbonylmethoxy)-*p*-*tert*-butylcalix[4]arene (12)

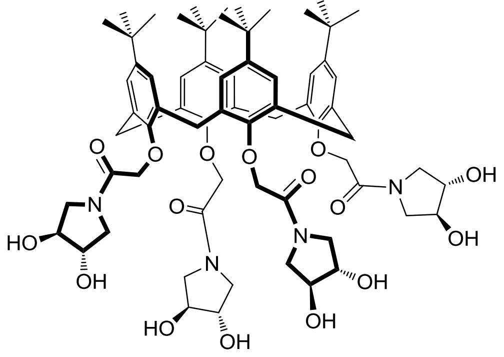

CsF (440 mg, 2.9 mmol) was added to a solution of **2** (390 mg, 0.18 mmol) in EtOH (23 mL) and the mixture was heated under reflux (and also the inorganic salt was dissolved) for 7 h. The clear solution was concentrated under reduced pressure to give an oil and the residual EtOH was evaporated off under high vacuum. The crude was triturated with CH_2Cl_2 and then let resting until precipitation occured. The yellow solution was carefully removed and the process was iterated until a clear solution was obtained. The white solid that was formed was washed with water (3 × 2 mL) and then dried under high vacuum to afford **12** (122 mg, 0.10 mmol).

White powder, 56% yield (CH_2Cl_2/MeOH = 10:1, R_f = 0.12); mp >260 °C (dec.); ^1H NMR (CD_3OD) δ 7.29 (s, 8H), 4.67–4.49 (m, 8H), 4.17–4.95 (m, 8H), 3.69–3.55 (m, 16H), 3.41 (d, 4H, J = 12.3 Hz), 3.30–3.23 (m, 4H), 1.18 (s, 36H); MS–ESI (m/z): $[M + Na]^+$ calcd. for $C_{68}H_{92}N_4O_{16}Na^+$, 1243.64; found, 1243.70; Anal.

calcd. for $C_{68}H_{92}N_4O_{16}\cdot 2H_2O$: C, 64.95; H, 7.69; N, 4.46; O, 22.90; found: C, 65.39; H, 7.60; N, 4.21.

Second generation calixarene dendrimer

25,26,27,28-Tetrakis[(3'*S*,4'*S*)-bis-(*tert*-butyl-dimethyl-silanyloxy)-pyrrolidinylcarbonylmethoxy]-*p*-*tert*-butylcalix[4]arene (3)

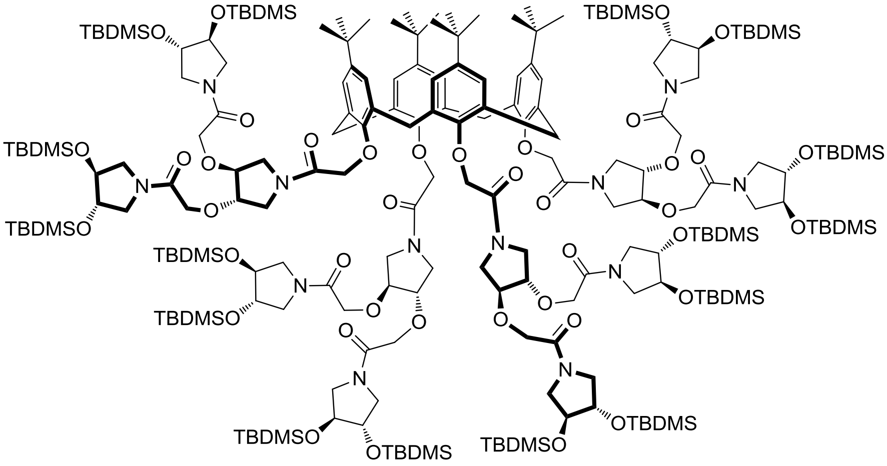

A solution of **11** (25 mg, 0.025 mmol) in CH_2Cl_2 (2 mL) was added to a suspension of **5** (77 mg, 0.08 mmol) in dry CH_2Cl_2 (2 mL) previously treated at 0 °C with DIPEA (140 µL, 0.80 mmol). After a few minutes a clear solution was obtained, and after 5 d at 30 °C under a nitrogen atmosphere a precipitate formed. CH_2Cl_2 (10 mL) was then added and the organic phase was washed with milli-Q water (6 × 10 mL). The clear solution was concentrated under reduced pressure to a dark oil, which was purified by FCC over neutral aluminium oxide (activated with 2% of water) using as eluent CH_2Cl_2/MeOH = 100:5 (gradient) to obtain target **3** (71 mg, 0.017 mmol).

White solid, 68% yield; ^{1}H NMR (400 MHz) δ 6.76–6.74 (m, 8H), 4.38–3.93 (m, 50H), 3.84–3.15 (m, 60H), 1.15 (s, 36H), 0.86 (s, 144H), 0.08 (s, 96H); ^{13}C NMR (very broad signals) δ 167.8 (s, 12C), 155.6 (s, 4C), 143.3 (s, 4C),

134.6 (s, 8C), 125.6 (d, 8C), 76.4 (d, 8C), 74.6 (d, 8C), 74.1 (d, 8C), 68.5 (t, 12C), 53.6 (t, 16C), 52.1 (t, 8C), 34.1 (s, 4C), 31.3 (q, 12C), 29.7 (t, 4C), 25.7 (q, 48C), 18.0 (s, 16C), −4.8 (q, 32C); IR (KBr): 2951 (m), 2930 (m), 2857 (w), 1464 (m), 1259 (m), 1108 (s), 836 (s), 778 (m) cm^{-1}; Anal. calcd. for $C_{212}H_{388}N_{12}O_{40}Si_{16}$: C, 60.70; H, 9.32; N, 4.01; O, 15.26; Si, 10.71; found: C, 60.43; H, 9.59; N, 4.36; $[\alpha]_D^{22}$ = −5 (*c* = 0.315, CH_2Cl_2).

4. Complexes of Compound 11 with Picrates

Alkali Metal Picrate Preparation

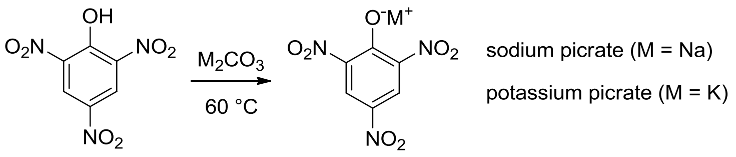

A commercially available saturated solution of picric acid (5 mL, about 0.3 mmol of acid) was carefully heated at 60 °C for a few minutes. (**Warning: Picric acid and its derivatives are unstable and may explode when dry!**) The desired metal carbonate was added in portions until CO_2 no longer forms, and once the pH was greater than 7 the solution was warmed and then cooled to 0 °C until a solid precipitated. The solid was filtered, washing with cold water, and stored wet and protected from light at −4 °C. The small quantities required for the tests were dried under high vacuum for 1 hour just before use.

Extraction Tests: Compound **2** (0.005 mmol), used as the ligand, was dissolved in $CDCl_3$ (1.0 mL) and then the insoluble metal picrate (large excess) was added. The mixture was stirred for 1 h (the colourless solution became yellow, indicating that the picrate had been partially dissolved) and then filtered into a NMR tube.

Sodium complex: Yellow solid (CH_2Cl_2/MeOH = 95:5, R_f = 0.20); ^{1}H NMR (400 MHz) δ 7.07 (s, 8H), 4.64 (d, 4H, *J* = 14.7 Hz), 4.36 (d, 4H, *J* = 12.3 Hz), 4.27 (d, 4H, *J* = 14.7 Hz), 4.02 (m, 4H), 3.96 (m, 4H), 3.68 (dd, 4H, *J* = 12.4, 4.1 Hz), 3.44 (d, 4H, *J* = 12.4 Hz), 3.40 (dd, 4H, *J* = 10.2, 4.1 Hz), 3.25 (d, 4H, *J* = 12.3 Hz), 3.04 (d, 4H, *J* = 10.2 Hz), 1.09 (s, 36H), 0.83 (s, 36H), 0.81 (s, 36H), 0.04 (s, 24H), 0.03 (s, 24H); ^{13}C NMR δ 167.8 (s, 4C), 151.0 (s, 4C), 147.9 (s, 4C), 134.5 (s, 4C), 134.3 (s, 4C), 125.7 (d, 4C), 125.6 (d, 4C), 76.4 (d, 4C), 74.6 (d, 4C), 74.1 (t, 4C), 51.7 (t, 4C), 51.3 (t, 4C), 34.1 (s, 4C), 31.2 (q, 12C), 29.9 (t, 4C), 25.7 (q, 12C), 25.6 (q, 12C), 17.8 (s, 8C), −4.7 (q, 8C), −4.8 (q, 8C); MS–ESI (*m*/*z*): $[M + Na]^+$ calcd. for $C_{116}H_{204}N_4O_{16}Si_8Na^+$, 2158.34; found, 2158.39.

Potassium complex: Yellow solid (CH_2Cl_2/MeOH = 95:5, R_f = 0.20); ^{1}H NMR (400 MHz) δ 7.06 (s, 8H), 4.60 (d, 4H, *J* = 14.4 Hz), 4.51 (d, 4H, *J* = 14.4 Hz), 4.48 (d, 4H, *J* = 11.7 Hz), 4.06 (m, 4H), 4.01 (m, 4H), 3.66 (dd, 4H, *J* = 12.1, 3.9 Hz), 3.51–3.46 (m, 8H), 3.24 (d, 4H, *J* = 12.1 Hz), 3.03 (bd, 4H, *J* = 8.9 Hz), 1.12 (s, 36H), 0.89 (s, 36H), 0.87 (s, 36H), 0.10 (s, 24H), 0.09 (s, 12H), 0.05 (s, 12H); MS–ESI (*m*/*z*): $[M + K]^+$ calcd. for $C_{116}H_{204}N_4O_{16}Si_8K^+$, 2173.31; found, 2173.27.

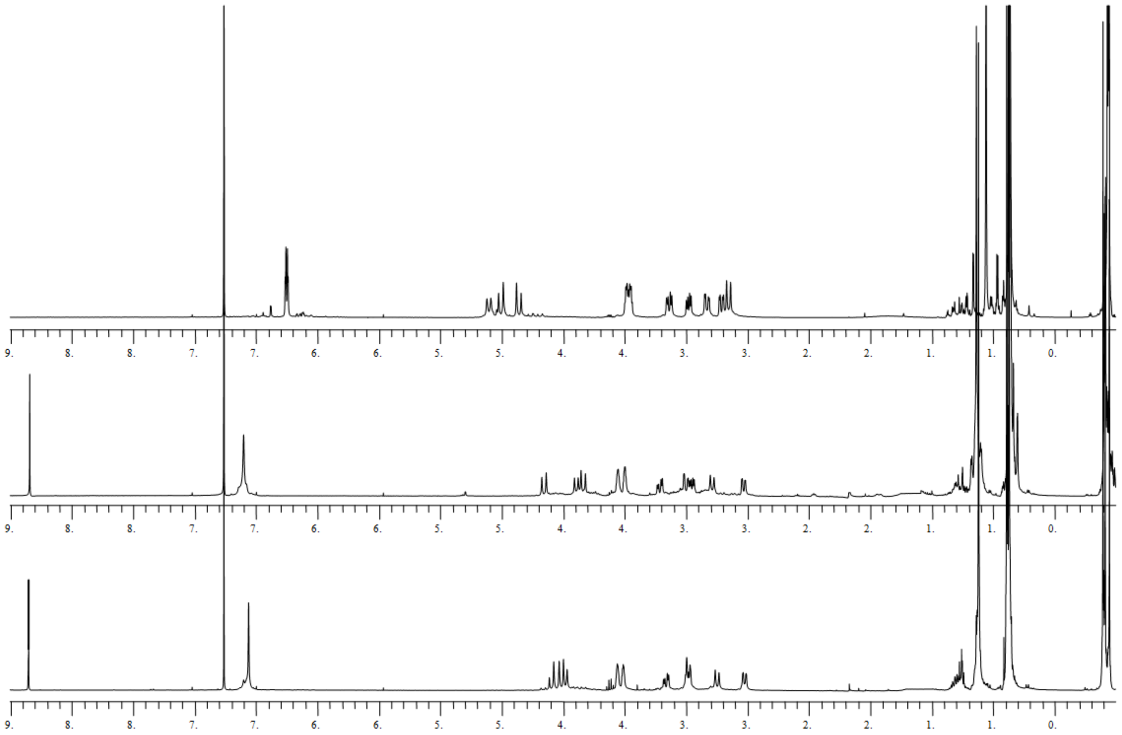

Figure S1: ^{1}H NMR full spectra of Figure 2: Free ligand **2**, sodium picrate complex, and potassium picrate complex (from top to bottom).

References

1S. Arakawa, Y.; Yoshifuji, S. *Chem. Pharm. Bull.* **1991,** *39,* 2219–2224. doi:10.1248/cpb.39.2219

2S. Arnaud-Neu, F.; Collins, E. M.; Deasy, M.; Ferguson, G.; Harris, S. J.; Kaitner, B.; Lough, A. J.; McKervey, M. A.; Marques, E.; Ruhl, B. L.; Schwing-Weill, M. J.; Seward, E. M. *J. Am. Chem. Soc.* **1989,** *111,* 8681–8691. doi:10.1021/ja00205a018

3S. Arduini, A.; Pochini, A.; Reverberi, S.; Ungaro, R. *J. Chem. Soc., Chem. Commun.* **1984,** 981–982.

4S. a) Molard, Y.; Parrot-Lopez, H. *Tetrahedron Lett.* **2002,** *43,* 6355–6358. doi:10.1016/S0040-4039(02)01386-2; b) Sanchez Peña, M.; Zhang, Y.;

Thibodeaux, S.; McLoughlin, M. L.; Muñoz de la Peña, A.; Warner, I. M. *Tetrahedron Lett.* **1996,** *37,* 5841–5844. doi:10.1016/0040-4039(96)01240-3

5S. a) Nagel, U.; Kinzel, E.; Andrade, J.; Prescher, G. *Chem. Ber*., **1986,** *119,* 3326–3342. doi:10.1002/cber.19861191112; b) Siedlecka, R.; Wojaczyńska, E.; Skarżewski, J. *Tetrahedron: Asymmetry*, **2004,** *15,* 1437–1444. doi:10.1016/j.tetasy.2004.03.015
